# Collectanea Medica, Consisting of Anecdotes, Facts, Extracts, Illustrations, Queries, Suggestions, &c.

**Published:** 1816-11

**Authors:** 


### COLLECTANEA MEDICA,

CONSISTING OF

ANECDOTES, FACTS, EXTRACTS, ILLUSTRATIONS, QUERIES, SUGGESTIONS, &c.

RELATING TO THE

*History or the Art of Medicine, and the Auxiliary Sciences.*

Quicquid agunt medici, nostri farrago libelli.

THE last number of the New-England Journal contains some very useful and learned remarks on a subject still involved in some obscurity, and on which we shall be thankful for any communications tions from those gentlemen of the faculty whose residence in the country enables them to gain information.

*On the Clavus or Ergot of Rye and other Plants;* by JACOB BIGELOW, M.D.

Many of the grasses and gramineous plants are subject to a disease, to which vegetable pathologists have given the name of *Clavus.* In this disease, one or more seeds are usually enlarged or elongated, and project from the spike or panicle to which they belong: they are of an irregular form, a light and brittle texture, a dark colour and unpleasant taste; and, as far as experiments have been tried, they are incapable of germination.

Of the different kinds of grain, rye appears to be most subject to this disease; wheat is often affected with it; barley and oats are said to be also liable to it. It likewise appears in the smaller grasses, both native and cultivated. I have observed it about Boston, in the fox-tail grass, *Alopecurus pratensis;* and the field reed, *Arundo cinnoides.* Mr. Curtis noticed it in the Flote Fescue grass, *Festuca fluitans.* Others have seen it in *Phalaris Canariensis, Avenor elatior, Lolium temulentum, Triticum repens, Phleum pratense, &c.*

Rye, if not the most subject to this disease, has at least most frequently attracted the notice of observers, in its diseased state. Spurred rye appears to have occurred not only in France and the midland countries of Europe, but also in all the climates from Sweden on the north, to Italy on the south, in both which countries dissertations relating to its prevalence and supposed effects have been published. Owing to the occurrence of certain epidemics, which for a time were attributed to this diseased grain, it became a subject of great interest to the community. Very different opinions were entertained in regard to its character, and more than thirty distinct treatises and memoirs were published at various times on this single morbid production of grain. In the United States it has lately come into notice as a medicine, and as a suspected cause of epidemic disease.

The circumstances which cause the generation of ergot in grain are obscure, and have led to many hypotheses. Agricultural observations, that have been made respecting it, seem to show, that low and moist grounds produce more spurred grain than soil which is elevated and dry; that the borders of fields are more subject to it than the central parts, and that new countries and grounds, lately cleared, more frequently give rise to it than fields which have been long cultivated. It is also said to abound most in rainy seasons.

A number of individuals have carefully watched the growth of ergotted rye in all its stages, but without coinciding in their reports as to its cause. Some have observed a viscid fermenting juice in the glumes, previously to the formation of the ergot; others have detected small *larvæ* of insects, which, being preserved, afterwards hatched into moths or butterflies. Among the more curious curious experiments relative to this subject is that of the Abbe Fontana,* who planted in his garden a number of single grains of wheat and of rye, and upon the top of each placed several grains of ergot. The result was a crop in which both the wheat and the rye were infected with ergot. As far as this experiment goes, it seems to indicate something like contagion in the disease, which may very possibly take place through the instrumentality of insects.

The prevention of ergot in grain must depend upon the cause producing it. If it is occasioned by insects, a remedy may possibly be found in sowing none but pure grain, or in exposing the grain to some process which may destroy the ovula infesting it. Formerly the ravages of an insect in France called the Corn butterfly were prevented by drying the wheat in an oven with a gentle heat before sewing it.† On the other hand, should the cause exist in the weather or climate, a partial remedy can only be hoped for in the selection of situations least adapted to its production.

A chemical analysis of spurred rye has been made in France by various individuals. It is stated to afford carbonic acid and hydrogen, a fœtid principle, a fixed oil in abundance, and a coal difficult to incinerate. Its extract putrefies very readily, and its colouring principle is fixed only by alkalies.

From one to two centuries ago, the spurred rye was suspected of producing certain epidemic diseases in different countries of Europe. These diseases are fully described in the numerous publications of the time, and in Encyclopedias and Medical Dictionaries ever since. As the propriety with which these epidemics

---

* *Journal de Physique par Rozier*, vii. 43.

The vegetable diseases produced by insects are so numerous and diversified, that scarcely any form of them need excite our astonishment. We every day see very singular excrescences on trees, shrubs, and plants, the size and substance of which vastly exceeds what is necessary for the support and habitation of the larvæ they contain. Of these, galls and oak apples are familiar examples, and by no means the most remarkable. It is one of the most astonishing instances of animal instinct and vegetable harmony, that an insect, by a puncture and deposit of its eggs, should so stimulate a plant, as to produce both a habitation and food for its offspring. Among the plants related to the grasses I have observed a rush, very common in the vicinity of Boston, *(Juncus polycephalos, Mx.)* the seeds or at least chaffs of which frequently grow to an enormous size, and are so altered in appearance that it would be nearly impossible to recognize the plant, were it not for the peculiar internal structure of its leaves. On opening these chaffs, or calyces, every one of them is found to contain the larva of an insect.

† Darwin's Phytologia, 363.

were attributed to the ergot has been discussed from a respectable source in the preceding pages of this journal, it is not necessary to dwell here upon the subject. It is sufficient to remark, that one of these diseases, the dry gangrene, has long ago disappeared, although the supposed cause has continued ever since: the other, a convulsive disorder, was successively ascribed to a variety of causes, one of the last of which was a weed among corn* (*Raphanus raphanistrum*), which obtained so much credit as to give a name to the disease (*Raphania*) in the Nosologies of Linnæus and Cullen. In New-England we have both the weed and the spurred rye, but no Raphania.

To ascertain whether spurred rye be really inimical to health and life, a great number of direct experiments upon animals have been undertaken by different individuals. These trials would doubtless have settled disputes relative to the properties of the article, had it not been for the wide and unaccountable difference which has appeared in their results.

M. Salerne, who published a memoir in 1748, states, that he gave the spurred rye, mixed with bran, to a pig for the space of a month. The animal declined continually, and at length died with marks of inflammation and gangrene in the intestines and liver. M. Read, in like manner, fed a pig with bran and ergot, which died gangrenous on the nineteenth day. He also killed flies in a few minutes with a decoction of ergot mixed with honey. The Abbe Tessier found the spurred rye fatal to a number of birds and quadrupeds, such as ducks, turkeys, and pigs. These animals died with external and internal marks of gangrene. The same author asserts that so great was the repugnance of animals to this vitiated grain, that many would die of hunger in preference to swallowing it.

On the other hand, there are writers who deny that effects so deleterious can have ensued from the use of ergot. Model fed pigeons for some time upon spurred grain, without any injurious consequences. Schleger made many experiments with it upon flies, dogs, fowls, a hog, a sheep, and other animals, without causing them any injury. He also states, that the powder applied externally to a recent wound proved perfectly harmless. Parmentier gave the ergot, both whole and made into bread, to pigeons, fowls, and a dog: he also took himself half a drachm a day, in the morning, fasting, for a week, and ate the flesh of the pigeons and fowls kept upon it, without the least inconvenience. Professor Murray† states the instance of two beggars who took every day an ounce of bread containing three drachms of ergot, for eight days, and remained all the time in good health.

From the discordant reports of the foregoing experiments, it is

---

* Amœnitates Academicæ, vol. vi.

† Apparatus Medicaminum, vol. v. where the preceding cases are stated.

obvious, obvious, that an impartial inquirer can draw from them very little satisfactory information. An extension of inquiries on the subject to other writers does not seem to remove the confusion, or lessen the number of contradictory inferences and results. On this account we may, without impropriety, set aside what has been said upon the subject in Europe, and attend only to the observations and inquiries into the properties of spurred grain which have been made in our own country.

Since its medicinal qualities began to excite notice, the ergot of rye has been continually observed, in various parts of the northern and middle states. I do not know that definitive observations have been made in regard to the kind of soil producing it, except that new grounds, or the soil of tracts newly cleared up, are more disposed to give rise to it than old fields, or those that have been long cultivated. Spring rye is considered by some to be more liable to it than that which is sown in the fall. Wheat appears to be affected by the same circumstances as rye; and considerable quantities of the ergot of that grain, brought from Vermont, have been offered for sale at the druggists' stores in Boston.

The spurred rye of this neighbourhood has a peculiar nauseous taste, attended with very little acrimony. When snuffed up the nostrils it does not prove sternutatory. Taken into the stomach, in some individuals, it occasions nausea in the dose of a scruple. A drachm excites greater nausea, and, in many instances, produces vomiting. It has not in general appeared to quicken the motions of the alimentary canal. Large doses have occasioned head-ach and temporary febrile symptoms.

The most remarkable effect of spurred rye, and that for which it has come into medicinal use in this country, is its power of acting, under certain circumstances, specifically upon the uterus. This property was first announced to the public by Dr. Stearns, of New York state, in 1807. It has been further investigated and discussed by Dr. Prescott, of Massachusetts, in a Dissertation published 1813, and by various writers in the different Journals and Gazettes. The use of this article in medicine is, to the best of our knowledge, an exclusively American practice, and if it is now introduced in any part of Europe, it must be from the publications of this country.*

It is now well ascertained, by the experience of a number of years, that the spurred rye given to parturient women has an unequivocal effect, in increasing the force of the uterine pains, and hastening the delivery of the child. This effect it sometimes fails to produce, but its failures are not more frequent than those to which almost any other article in the materia medica is liable. Its character as a medicine is so well established, that a majority of

* Dr. Prescott's Dissertation, read before the Massachusetts Medical Society, has been republished in England, and translated into French by M. Charbonnier.

practitioners in Boston, and, probably, throughout this state, are in the habit of employing it in cases where a medicine of this sort is indicated. Under proper regulations, it may be considered a valuable addition to the present stock of medicinal agents.

When given prematurely, or under improper circumstances, spurred rye has proved injurious to the mother, and still more frequently to the offspring. The first cautionary hints on this subject were given in this Journal more than four years ago. When administered at too early a stage, or while considerable obstacles to delivery exist, it creates unnecessary suffering to the mother, and endangers the child's life. The principle circumstances which contra-indicate its use, may be found in the various notices that have been published on the subject. Among them may be mentioned earliness of the stage, rigidity of the soft parts, any unfavourable conformation, or any presentation that requires changing.

From its power of stimulating the gravid uterus, practitioners have been led to try its effects on the uterine system under other circumstances. In the disease of amenorrhea it has been given to a considerable extent, with various success in the hands of different physicians.

Dr. Thacher mentions one or more cases in which the obstructed catamenia were restored by a small quantity, a drachm only, of this medicine. Dr. Prescott and some others have mentioned cases of its failure. I have given, in two instances, an ounce in substance, in the course of a week, without effect. But the following statements, communicated to me by a medical friend,* appear to contain more extensive trials, both of the efficacy and safety of the medicine, than any I have met with. His statements are as follow:

"The case in which I gave the greatest quantity of ergot was one of amenorrhea, and happened about three years since. The quantity of the medicine taken was six ounces. The patient was furnished with the ergot at two different times—two ounces the first, and four the second time. The medicine was prepared with a quart of water to an ounce of ergot, boiled down to a pint. The first quantity, as directed, was taken in five days; but the second, from the great solicitude for relief, was taken in less than four days, so that more than an ounce was taken in a day. The patient was relieved: no unpleasant effects occurred at the time, and she has remained in perfect health ever since.

"The next case of magnitude was Mrs. T. She has taken the ergot for amenorrhea at three different times, at the rate of four drachms a day. An ounce relieved her the first, the same quantity the second, and two ounces the third time. No dangerous symptoms happened to her on any of the occasions, and she has enjoyed as good health since as she ever did before.

---

* John Randall, M.D.

"I have

"I have given half an ounce per day to another person for four successive days, without any ill effects, but without giving the relief desired.—Four other persons, by my direction, have taken an ounce each in the quantity of half an ounce per day, with perfect relief and without injury.

"The symptoms produced in the seven preceding cases, as far as I have learnt them, were head-ach, increased heat of body, and occasional pain in the hypogastric region. Probably sickness of the stomach may be added, although I have not always noticed it."

The foregoing cases sufficiently show that the spurred rye may be given to a very considerable extent, without other injury than the temporary inconvenience which occurs at the time of its exhibition. It would also seem entitled to the reputation of an emmenagogue, at least as much so as many remedies of that uncertain class which are now in use.

In regard to the preparations of this medicine, the infusion and decoction appear to extract all its active properties. The latter operates more speedily than where the crude powder is given. The dose usually administered to women in labour is from ten grains to half a drachm in decoction, to be repeated, if necessary. Some practitioners begin with a drachm. Patients who have taken this last amount, frequently vomit before or after delivery.

The ergot of *wheat* has been the subject of a few trials, which serve, in some degree, to establish its affinity to that of rye. Its taste is equally nauseous, and somewhat more unlike that of the original grain. I have seen it occasion nausea in the dose of a scruple, and vomiting when a drachm had been given. In some cases of labour, it has evidently increased the uterine efforts; in one it produced no effect.

In regard to the agency of spurred grain, in generating the epidemics, which, of late years, have appeared in various parts of the United States, the question is too extensive to be hastily decided on by an individual. The suspicions which have been cast upon this article no doubt had their origin in the terrors which it formerly excited in Europe. It is probable, if the ergot has any instrumentality in generating disease, that it requires, to say the least, the assistance of some co-operating cause; since we have seen that it can be taken to a greater extent than it ever would be for the purposes of food, without any permanent injury; and also since we have sufficient evidence, that it has existed in this country long before the diseases that have been laid to its charge, and when people were not led to avoid its use by any knowledge of its medicinal powers.

Since, however, the ergot, in large quantities, would prove injurious in food, from its medicinal agency, and in smaller quantities would impair the taste and nutritive qualities of bread, it is always desirable that it should be separated, as far as possible, from from the sound grain. This may be accomplished, to a sufficient degree, by care in the process of winnowing. The grains of ergot, being lighter and generally larger than the sound grains, are either detained by the sieve, or blown away by the wind. Those who collect ergot for the druggists have told me that they procure it in larger quantities from the chaff than from the grain. It is probable that, in all cases, if the winnowing be properly repeated, the grain will, without difficulty, be made sufficiently pure for use.

---

*Observations on some Disorders of the Eyes;* by John C. Warren, M.D. Professor of Anatomy and Surgery in Harvard University.

*Rheumatism of the Eyes.*—A gentleman was attacked with pain in the head, nausea, and inflammation in the right eye. I bled him to the amount of twenty ounces, and directed an emetic. The complaint in the eye increasing, he was ordered warm fomentations, blistering, and a strong purgative. On the fourth day, I observed a spot on the cornea connected with a fasciculus of vessels on the conjunctiva, and, being fearful of the effect of the consequences on his vision, I excised the vessels of the conjunctiva. In a few hours the spot, together with a general opacity of the cornea, connected with it, had disappeared. Soon after, he complained of a pain and swelling in the right knee, for which he was directed hot fomentations and affusions of tepid water. These applications gave a partial relief; but this relief was immediately followed by an increase of pain in the eye, and a return of opacity in the cornea. A great number of leeches were applied to the eye, a warm solution of sulphate of zinc, and the vicinity of the eye was blistered. The pain in the eye ceased; the redness and some degree of opacity continued. Immediately on this, the knee swelled, and became excessively painful. Now, the complaint remained obstinately fixed in that part, while at the same time the other knee and the wrists swelled. After these occurrences, the local applications were, in a great measure, suspended, and recourse was had to general remedies. Mercury, opium, antimony, warm bathing, all carried to the farthest point of administration, gave him no permanent relief. Blisters were followed by some mitigation of the pain. After two months he tried the wine of hellebore, and gradually increasing the dose to forty drops every three hours, he was completely poisoned by it. His whole body swelled, and was covered with an eruption; the tongue was perfectly dry, and so swelled as scarcely to be moveable; but he complained most of a distressing load at the stomach. The last symptom was relieved by an emetic. The others gradually subsided under the use of purgatives and warm bathing; and, as soon as the swelling of the body would permit him to make an exertion, he found his knees flexible, and without pain. The symptoms of disease had now completely subsided, except a stiffness of the knee joints, which disappeared gradually.—A year after he had another another attack of inflammation in the eye, with diurnal paroxysms of pain. After a copious bleeding, he was relieved in about ten days, under the use of a solution of the oxyd of arsenic and some blisters.

A gentleman, of feeble constitution, whom I had cured of a severe urethral complaint some years before, was attacked with a pain and swelling of one knee. Some trifling remedies were employed. Three days after he was affected with the symptoms of continued fever, which lasted about twenty days, and nearly proved fatal. During his illness the knee was slightly swelled. As he recovered the swelling increased, and was attended with pain. He rubbed the knee with brandy, and the next day had an inflammation of the eyes, which lasted about ten days. The only applications were blistering, and a solution of the acetite of lead with laudanum. During this inflammation, the pain in the knee was not troublesome; but no sooner did it subside than the pain in the knee became excessive: the solid parts of the joint formed a hard swelling, on which the finger made no impression. The muscles of the neck became stiff and painful, and one of the wrists swelled. After some vapour baths, fomentations, and leeches, and three months of extensive blistering about the knee, he was relieved of pain and swelling. The motion of the joint was restored by frictions and exercise.

A gentleman came to me from the country with a severe inflammation of the left eye, attended with opacity of the cornea sufficient wholly to obstruct the admission of light. He experienced so little inconvenience from this inflammation that he had not thought it necessary to protect the eye by any covering. He informed me that the inflammation and opacity would subside in a few days, and his eye would be as sound as the other; that he had been subject to these attacks about ten years, and that his object in applying for advice, was principally with a view of ascertaining whether any method could be devised of preventing the recurrence of the attacks, as, though he was not uneasy about the actual opacity, he was fearful it might, at some period, become fixed. Being suspicious that his case might be of the nature of those stated before, I enquired whether he was subject to rheumatism, and received for answer that he had been so from about the time that his inflammation of the eye commenced, and that when his eye was inflamed, the limbs were commonly easy.

During the past winter, rheumatism has been unusually prevalent, whence I have had an opportunity of observing a number of cases of rheumatic ophthalmia, though less distinctly marked than those mentioned above. One circumstance was common to all—that the disorder of the eyes was but little affected by the most powerful local applications, and required the use of general remedies to affect a cure.*

*Weakness*

* It is not my intention to make general remarks on this complaint;

*Weakness of Sight.*—Having been often defeated in attempts to cure a defect of vision, arising from great sensibility to light, I took the earliest opportunity of trying the depleting practice recommended by Mr. Stephenson. This did not succeed so well as I wished; and a little reflection served to convince me that the application of five or six leeches to the eye could not be expected to produce a change in the state of a chronic disease, supposed to reside in the internal coats of the organ. A more liberal use of the same remedies has been followed with very satisfactory effects.

A lady, in the prime of life, of firm constitution, subject to severe fits of periodical head-ach, had been for two or three years suffering from such a sensibility to light, as at last to prevent her employing the eyes in any labour or amusement. She had used a multitude of remedies without advantage. At first I took a large quantity of blood from the arm, directed permanent blisters behind the ears, frequent purgatives, and a regular mercurial course. Evidences of constitutional affection from mercury were kept up about six weeks, without any advantage. The head-ach being now very regular and very troublesome, bleeding was repeated, and large doses of Peruvian bark were given at first, and then a solution of arsenic, with the expectation of curing the head-ach, and, possibly, the disorder of the eyes at the same time, as they might depend on the same cause. The solution of arsenic was continued a long time, without any good effect. Not being able yet to obtain leeches, I directed a solution of the extract of hyoscyamus to be applied every other evening. Its immediate effect was a dilatation of the pupil and increase of suffering; but the day following the application was always more comfortable than usual. No material change in the complaint was effected, however, and the patient became discouraged; yet was still ready to try any thing which promised relief. As soon as the season admitted the procuring a sufficient number of leeches, the application was commenced. On the first day twenty leeches were applied, on the second thirty, and on the third twenty-five. The leeches were large and voracious. One of them, weighed before and after being applied, was found to have taken three drachms of blood. After an intermission of two days, the application was

---

plaint; but it may be well to note, that, among other appearances during the winter, I have met with three cases of paralysis of one side following rheumatic affections—one of severe spasmodic disease of the intestines; one of sudden delirium, by a metastasis from the back to the brain; two or three of inflammation of the pleura, which I was inclined to consider of the same nature. These last were very distressing in one instance, while in others, although there was a considerable bloody expectoration, the other symptoms were remarkably mild. The cases of paralysis were all cured by vesication; yet not so completely as to restore the natural strength of the affected limbs.

again again made; thirty were applied on the first day, thirty-five on the second, and forty-seven on the third. Two more days were allowed to elapse, and then about the same number was again applied during three successive days. A slight degree of debility was now experienced, and the eyes felt a little relieved, so as to encourage a continuance of the leeches. After a week, the same course was repeated; then an interval of about three weeks elapsed, and another application was made. The eyes were now mending rapidly. The leeches were discontinued; a seton made; a vegetable diet and exercise in the air recommended, the eyes being shaded; and, as the patient was desirous of using the solution of hyoscyamus occasionally, this was permitted. In a very gradual manner the healthy state of the eyes was restored, so that at the end of three months from the first use of the leeches, I had the pleasure of seeing her perfectly cured, and she has now continued well about two years.

A young lady, of delicate constitution, requested my advice for debility of vision, and extreme sensibility to light. She had been so long afflicted with this complaint, and had used so many remedies, as to have become almost hopeless of relief. Before using leeches, I was desirous of making trial of the extract of hyoscyamus or of stramonium. Blisters were directed behind the ears, and a grain of calomel given every night, and a warm solution of one of these narcotic extracts ordered twice a-day. No benefit being derived from these applications, the use of leeches was resorted to. She made a few trials with eighteen or twenty, and afterwards increased the number to sixty in one morning. This last application caused a degree of faintness, and was followed with a sensible change for the better. Permanent blisters were then applied, and continued about two months, during which time her sight was much improved, and her situation rendered very pleasant, compared to what it had been. The blisters in this case were useful probably, but ought not to receive great credit, as they had frequently been employed before the leeches without any advantage.

Mr. R., a gentleman of about thirty, had been subject to a defect of vision for a number of years. At the time I saw him he was unable to read more than a few lines, before a sudden confusion or blur spread itself over every object, and entirely defeated his efforts to read. No leeches were to be had, and he was therefore ordered a course of brisk purgatives, blisters behind the ears and neck, and a course of the muriate of mercury. Not the slightest benefit was perceived from this course during the three months he followed it. Leeches were then applied, about thirty at a time for three or four successive days. Their beneficial effect was immediate. He was relieved on the first application; and, after applying them occasionally for three or four weeks, he was so well satisfied with his condition as to omit all further remedies.

I have known a single application of a great number of leeches effect a remarkable and permanent relief of this complaint.

*Dislocation of the Crystalline Lens.*—The escape of the crystalline from its capsule is an accident now and then mentioned; but has not, that I know of, been very distinctly spoken of. The first case of the kind which I noticed was in a man of about forty, who was suddenly attacked with violent pain in his right eye, without any external cause. With these pains he had been distressed for many weeks, when I first visited him. On inspecting the eye, the lens was seen to be opaque and near to the cornea; the conjunctiva was inflamed, yet less than might be supposed. I opened the cornea with a cataract knife, and with some difficulty raised it from the iris. After this, the crystalline was dissected from the iris, to which it strongly adhered, and extracted from the eye. The subsequent inflammation was not remarkable, the wound healed slowly, the pains in the eye were relieved, and have never recurred.

Mr. G. was attacked with a severe pain in one of his eyes, which, on being examined, exhibited an immoveable pupil, in which lay the crystalline lens, having its edge projected through the opening. It was proposed to Mr. G. to extract the lens; but, on coming to do the operation, his resolution failed, nor could he, at any subsequent time, collect courage to support it. A chronic inflammation of the eye was the consequence, which continued to trouble him to the day of his death; and this event was probably accelerated by the perpetual irritation of his eye.

A lady of irritable habit, very subject to indisposition, had an inflammation of the eye, the cause of which was obscure. After many observations, it appeared that the pupil of this eye was immoveable, and slightly dilated, and the vision of the organ lost, yet the crystalline lens retained its transparency. After two years, the lens had become completely opaque. She is subject to pains on that side of the head, and to an inflammation of the eye recurring about once a month. Being very alarmingly threatened with ulceration of the lungs during three or four months, she continued for that time exempt from inflammation; but no sooner were the dangerous symptoms dissipated, than the inflammation returned. In truth, she is of opinion that, whenever her eye is disordered, her general feelings are best. Still it is probable that the result of this irritation must be, on the whole, injurious. She does not, however, suffer sufficiently to render the extractions of the lens very important.

In another instance, the lens pushed through the iris, pressed on the cornea, and brought on a painful state of the eye, which continued nearly three years; then inflammation, suppuration, and ulceration in this membrane. Finding a small opening in the cornea, I enlarged it, and removed the lens, which was found to be hard. The extraction of the lens, and discharge of pus, was not, however, followed by the relief which was expected, for the pain continued with dreadful severity. On examining the eye, I found a very firm opaque substance lying in the wound of the cornea, which I removed, and then threw warm water into the cavity of the the eye, with the intention of removing any other substance of the same kind. This gave a temporary relief, but the pain soon returned. On reflection, I was convinced that the disorder was kept up by the thickened and almost solid vitreous membrane. I therefore made a crucial incision across the cornea, even into the sclerotica, extracted the remainder of this substance, and completely relieved the patient. The wound afterwards healed, and he now wears a glass eye with great satisfaction.

A gentleman, receiving a fall, struck the edge of the orbit of the left eye. On the next day, a great inflammation of the right eye appeared; and, on examining it, I found the pupil excessively dilated, and the vision of the eye lost. In six or eight weeks the violence of the inflammation subsided, and he is now very comfortable, although the eye is weak, and requires to be protected from the air. The pupil remains dilated and immoveable. No degree of vision returns. I proposed to extract the lens, but at present he declines the operation.

A woman was suddenly seized with a violent pain on the right side of the head, followed by vertigo, delirium, fever, and symptoms of phrenitis. The inflammatory symptoms disappeared after a few days; but the pains continued, and she discovered that the sight of the right eye was lost. About the tenth day I saw her: found the eye much inflamed, very tender, and painful; and the pupil completely dilated and insensible to light. She was bled a pint; and, on the succeeding days, had blisters behind the ears, very powerful purgatives and opiates at night. Finding her sufferings were not alleviated, nor the eye less inflamed, I made a puncture in the cornea, introduced a probe, and found the crystalline lens pressing, as I expected, on the cornea. The incision was then enlarged with scissors, and the crystalline lens extracted. This operation was followed by some days of ease; but then the pains returned with as much severity as ever. After this, finding she was not permanently relieved by bleeding, blistering, nor a slight salivation, and that the vitreous humour was projecting through the wound in the cornea, I removed the protruded part of this substance. Still, however, the pains continued excessive; on which I passed a cataract knife into the cornea, made a large crucial incision, gave vent to a considerable part of the discoloured vitreous humour, and thus relieved her. Since then she has been recovering.

The cause of spontaneous dislocation of the crystalline lens is not known. This accident must arise from a change in the lens itself, or in its capsule. In some of the cases I believe the lens has been evidently disordered, before the symptoms of dislocation took place, which would make it probable that the cause of the disorder resides in the lens; and I recollect to have dissected a decayed eye, in a subject examined for another purpose, in the cavity of which was found nothing but a little watery fluid and a stony crystalline lens. In this case there cannot be much doubt, that the petrified lens had caused absorption of its capsule, and thence escaping, had pressed on other parts, and brought on inflammation and ulceration of the eye, followed by a discharge of the contents of the globe, and a condensation of its coats into a single membrane.—*New-Eng. Journ.*

---

The following case very much confirms the doctrine of Spurzheim on the fibrous structure of the brain.

*A Case of Water in the Head, unattended by its usual Symptoms; with Observations.* By W. Heberden, M.D. F.R.S. Fellow of the Royal College of Physicians, &c.

In adverting to the circumstances of the following case, I have thought it might not be unworthy the attention of the College of Physicians; for, though I have as little respect as any man for facts, merely because they may be extraordinary, yet, when such facts can in any way contribute to perfect our knowledge, or to correct our judgment, they acquire a degree of value in proportion as they promote these ends. This is not the place to teach the common symptoms of common disorders. But it may not be without its use, in the investigation and treatment of the most common, to know what alterations of structure may take place, unattended by their usual signs, or what signs may be manifested independent on their usual causes. If the pride of theory be sometimes humbled, the conclusions of modest judgment can at least never be impaired by real knowledge.

I shall first give an account of the appearances after death, in the words of an able surgeon who conducted the examination, and shall afterwards state what I had observed while the patient lived.

"On attempting to remove the calvaria, the dura mater adhered with so much firmness, as to split into layers, rather than quit its connexion with the bone. The arachnoid membrane was thickened, of a milky colour, and much elevated by the subjacent fluid, which pervaded the cellular structure of the pia mater, to the amount of four ounces. The substance of the brain was healthy. The ventricles were greatly enlarged, and contained about eight ounces of transparent water. The plexus choroides was pale and void of vessels, containing many minute vesicles resembling hydatids, and one solid tumour, which appeared to contain the same calculous matter which is met with in the pineal gland. The internal carotid and basilary arteries, with many of their primary branches, were ossified. In the chest, the lungs were found firmly adhering to the parietes; and some small tumours, containing calculous matter, probably phosphate of lime, were dispersed through their substance. In the heart, the valves of the left auriculo-ventricular opening were partially ossified, those of the aorta completely so, and small depositions of bony matter were found on the tendinous portions of the carneæ columnæ. The coronary artery was ossified through its whole extent. The aorta, soon after after emerging from the ventricle, formed a true aneurismal pouch, consisting of a dilatation of the coats of the artery, without any rupture of its internal lining. The descending thoracic and abdominal aorta, with all their primary branches, were converted into cylinders of bone, as also were the external and internal iliacs.

"The intestines were, in many places, firmly adhering by their peritonæal surfaces, apparently the result of former attacks of inflammation. At each abdominal ring there was a small hernial sac.

"The liver was healthy; the spleen covered with a bony deposition, between its peritonæal covering and tunica propria.

"The bladder contained some turbid urine; and a small spot near its neck had a blackened appearance. The prostate gland was enlarged. The kidneys were healthy. In the left testicle there was a collection of water."

Such was the account which I received from Mr. Henry Earle. The following circumstances contain the sum of my knowledge respecting this patient.

Mr. W. was an old man: the person with whom he lodged believed him to be some years turned of fourscore. I had attended him occasionally, but not often, during the last ten years of his life. From the time when I first saw him (I know not how much longer), he had been exceedingly deaf, so that it was almost impossible to make him hear, and I was obliged to write down all that I would ask him, or say to him. But for this defect, he appeared of a sound constitution, and free from the usual infirmities of advanced age. When he consulted me, it had been upon occasion of some temporary feverishness, expectoration, or other transient indisposition, and once or twice on account of giddiness, for which he was cupped with advantage. But I never saw him under any serious illness till about six weeks before his death. I was then sent for, and was informed by his landlady, that she apprehended he must have had a fit in the night; that he had scrambled or fallen out of bed, and seemed confused, with great weakness, particularly in his legs. He had collected himself before I got to him, but was feeble, had a quick pulse, and had lost all relish for food. He, however, told me distinctly that he had no giddiness, nor pain in his head, or elsewhere. In a few days his symptoms went off; and, after ten days or a fortnight, I took my leave. At that time his appetite had returned, his pulse was equal and natural, his breath perfectly free, his voice strong, and his understanding and memory clear and good. There seemed to be no longer any remains of illness, unless that his legs, though not so weak as before, were still weak. From that time I had called at his house once, and was informed that he continued as well as usual; but I did not see him till the evening of February the 12th, when I was again desired to visit him, and found him dying. I was told that he seemed indisposed all day, but had given the people about him no alarm till they sent for me. He was lying perfectly still in his bed, apparently senseless; his pulse could hardly hardly be felt, and he breathed like one who was expiring. He died about five hours afterwards.

Before we take leave of this case, it may be worth while to offer some observations by way of illustration; for, without attempting to explain every particular, we may find reason, from analogy, to admit certain principles, which are, in a general view, applicable to the subject of this paper.

When it is considered how minute a puncture, what a gentle pressure of the brain is often felt through the whole frame, producing sometimes irregular and excessive actions, a painful sensibility to light, noise, and all those ordinary exciting causes, which should only awaken us to a pleasing consciousness of our existence; or, on the other hand, benumbing all the natural faculties, so that the mind, instead of that delightful energy and activity with which it uses to soar into unknown worlds, is reduced to a calamitous state of torpor, or fatuity, while the body scarce drags along its own unfeeling limbs; when, I say, these are the ordinary and well-known effects of pressure upon that delicate organ, it may well excite our surprise that so large an accumulation of fluid (not less than three quarters of a pint), and consequently so great a compression, should ever be sustained with little or no manifest inconvenience.

The reason of this may, perhaps, be suggested by the very gradual manner in which it may be supposed to have been collected, and in that wonderful power, with which every living body is endowed, of accommodating itself to new circumstances. For, if only time be given for the neighbouring parts to contract or enlarge themselves, as the case may require, it is difficult to set any limits to this faculty.

We see every day what great exertions the human body is capable of, by regular education and discipline; so that, on comparing together the limbs of a hardy workman and one of sedentary or effeminate habits, we might be inclined to class them as creatures of different species, at least as distinct as the different races of dogs, or of horses, or other brute animals. It is less surprising, that the mind of man, so subtile in its nature, and so active in its principle, should show an endless variety in its capacity and exertions, sometimes animating a Newton, sometimes sleeping in an ideot. But even in parts which one might expect to be less liable to fluctuation, and more intimately connected with the constitution of our frame, we still observe the same faculty of adopting habits, it may be, the most contrary to nature. The use of tobacco and spirituous liquors is so familiar as scarcely to excite attention; but what shall we say of opium, and other poisons, which we know may gradually be increased to quantities which would presently be fatal to persons unaccustomed to their use. The very organs of sensation partake of the same disposition, so that the roar of cannon is not more distressing to one ear than the want of noise in a calm and still night is to another. It is remarkable, too, with what acuteness one sense can supply the place of of another; which shows, at least, to what an increased degree of perfection they are capable of attaining. The same thing is, perhaps, yet more conspicuous in the obliteration of certain parts of the body itself, as in the operation for the aneurism, after which the blood finds new channels opening for its conveyance, in proportion as they are wanted.

Many other examples might be produced; but it is apprehended these hints are quite sufficient to awaken a recollection of the very great alterations which every living body is enabled to undergo; and, I conceive, it is no extravagant idea to extend to the brain the same principle of self-preservation, which is certainly possessed by so many parts of the body more within our observation.

It is uncertain how far this gentleman's deafness ought to be attributed to the altered condition of the brain. The supposition appears to be sufficiently probable; and, if it be just, it furnishes an argument for believing that the disorder had been many years forming. I witnessed the deafness for ten years, and I know not how much longer it may have existed.

The brain, from its situation, is not to be got at without considerable trouble, and considerable deformity to the corpse. It is, therefore, not often made the subject of examination, unless the symptoms preceding death have rendered it probable that something more than ordinary will be found. Hence accumulations, of the kind that I have described, may very well be supposed to exist in many subjects where they are never discovered.

The case I have related is not the only one upon record. Upon turning to Morgagni, I find one very similar, which, being short, I shall take the liberty to transcribe. It is probable that a more diligent search would have furnished me with others of the same kind.

"Senex prope annos octoginta natus, olim, quod cicatrices ostendebant, tibiarum ulceribus, nunc fœdis in cute fere universa pustulis, affectus, in Bononiense S. Mariæ de Morte nosocomium sub noctis initium recipitur. Arteriarum pulsus non frequentes quidem illi erant, sed vi parum firma, eaque inæquali, neque in utroque brachio æque manifesta. Lucebant oculi, intentique erant, et quasi diversa tuentes. Interrogatus negat caput dolere, aut grave esse, aut somnolentum. Vomuisse se ait, idque lingua, ut videtur, titubante. Mens tamen, et sensus, et movendi facultas constant. Noctu sensim gravior fit: itaque moritur postridie mane. Ventre aperto sana omnia inventa sunt, nisi quod erant multo magis, quam solent, madida; hepar autem, subalbidum et duriusculum, ejusque vesicula referta subnigra bile: colon denique intestinum, si id quoque huc censes attinere, ad crassitudinem pollicis sub ventriculo contractum. Hora erat a morte undecima, cadaver autem in aperto aëre jacuerat, eoque frigido, medius enim erat mensis December A. 1705, intestina tamen adhuc calebant. Pulmones undique erant pleuræ affixi, a qua, dum sinistro in latere avelleruntur, aqua prodiit, quæ ubi stagnasset pro certo non potuit cognosci. In corde, ut alibi quoque, sanguis fluidus. Capite

 abscisso, abscisso, aqua de maximo cranii foramine destillabat; et sane ubique intra cranium fuit, præsertim vero sub tota meninge tenui, per quam, spumosæ salivæ instar, bullulis videlicet passim admistis, translucebat. Plexus Choroides vesiculas aliquot aqua distentas habuere; ipsi tamen ab ea aqua, quæ in ventriculis inventa est, minime albicabant. Cerebrum laxum erat: Pituitaria glandula quasi nulla."*

It will be observed, that the subjects of both these cases were old men. The quantity of water, in one case, amounted, as we have seen, to twelve ounces; in the other it does not appear to have been measured, but it is said to have trickled from the base of the skull, and to have been found in every part of the brain. In both cases, the interior membrane of the brain was distended with fluid; and the plexus choroides, in both, contained vesicles full of water. Again, both patients had been free from pain, heaviness, drowsiness, or any other ordinary effect of pressure on the brain; and in both, the senses and understanding had been unimpaired.—*Med. Trans. of the London College.*

We may add that Morgagni's subject had only ceased to breathe for eleven hours, and that the intestines were still warm. Their general moisture probably arose from their condensed exhalation, the body having been deposited in a cold place. There does not appear to have been, in the cavity, any collection of fluid which could be imputed to transudation after death. Hence the fluid found in the brain could not be imputed to that cause.—Edit.

